# Coordination of the Arc Regulatory System and Pheromone-Mediated Positive Feedback in Controlling the *Vibrio fischeri lux* Operon

**DOI:** 10.1371/journal.pone.0049590

**Published:** 2012-11-13

**Authors:** Alecia N. Septer, Eric V. Stabb

**Affiliations:** Department of Microbiology, University of Georgia, Athens, Georgia, United States of America; Auburn University, United States of America

## Abstract

Bacterial pheromone signaling is often governed both by environmentally responsive regulators and by positive feedback. This regulatory combination has the potential to coordinate a group response among distinct subpopulations that perceive key environmental stimuli differently. We have explored the interplay between an environmentally responsive regulator and pheromone-mediated positive feedback in intercellular signaling by *Vibrio fischeri* ES114, a bioluminescent bacterium that colonizes the squid *Euprymna scolopes*. Bioluminescence in ES114 is controlled in part by *N*-(3-oxohexanoyl)-L-homoserine lactone (3OC6), a pheromone produced by LuxI that together with LuxR activates transcription of the *luxICDABEG* operon, initiating a positive feedback loop and inducing luminescence. The *lux* operon is also regulated by environmentally responsive regulators, including the redox-responsive ArcA/ArcB system, which directly represses *lux* in culture. Here we show that inactivating *arcA* leads to increased 3OC6 accumulation to initiate positive feedback. In the absence of positive feedback, *arcA*-mediated control of luminescence was only ∼2-fold, but *luxI*-dependent positive feedback contributed more than 100 fold to the net induction of luminescence in the *arcA* mutant. Consistent with this overriding importance of positive feedback, 3OC6 produced by the *arcA* mutant induced luminescence in nearby wild-type cells, overcoming their ArcA repression of *lux*. Similarly, we found that artificially inducing ArcA could effectively repress luminescence before, but not after, positive feedback was initiated. Finally, we show that 3OC6 produced by a subpopulation of symbiotic cells can induce luminescence in other cells co-colonizing the host. Our results suggest that even transient loss of ArcA-mediated regulation in a sub-population of cells can induce luminescence in a wider community. Moreover, they indicate that 3OC6 can communicate information about both cell density and the state of ArcA/ArcB.

## Introduction

Many bacteria regulate gene expression by producing and sensing pheromones. Because these signals can accumulate as culture density increases, pheromone-mediated responses often depend on high cell densities, giving rise to the term “quorum sensing” [1]. However, in many systems pheromone signaling is not simply a function of cell density. Instead, both synthesis of pheromones and responsiveness to them are often context dependent. Environmentally responsive regulators control expression of many pheromone synthases and/or their cognate receptors, rendering such signaling dependent on other parameters in addition to cell density [2]–[27]. Moreover, pheromone signals often stimulate an increased rate of their own synthesis [28]–[39]. This positive feedback can mean that even at the same cell density, the concentration and synthesis of a pheromone are partly a function of whether the system has recently been in a stimulated state.

Combining context-dependent regulatory control over pheromone synthesis with pheromone-mediated positive feedback has profound functional implications. Positive feedback can amplify the effects of other regulatory inputs that modulate pheromone synthesis, and the relative strengths of a regulatory input and positive feedback will affect communication, particularly in a population that spans a heterogeneous environment. If positive feedback is strong, a subpopulation of bacteria experiencing an environment that favors pheromone production might elicit a population-wide response, even in cells that would otherwise remain uninduced given their distinct environmental context. If positive feedback is weak, local environmental context becomes a more defining determinant of whether a pheromone system is induced. Understanding how bacteria integrate pheromone sensing, environmentally responsive regulation, and positive feedback to coordinate group responses requires model systems with pheromone-mediated behaviors that are easily observable and induced in natural environments.

**Figure 1:**
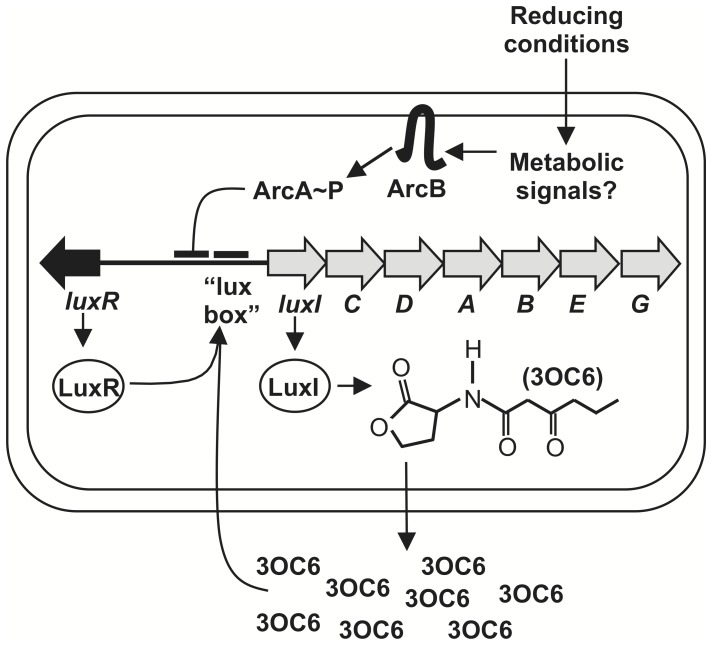
ArcA/ArcB and LuxR-LuxI-mediated regulation of bioluminescence in *V. fischeri*. LuxI synthesizes 3OC6, a diffusible pheromone that upon reaching a sufficient concentration combines with LuxR. 3OC6-LuxR binds to the “*lux* box” and stimulates transcription of the *luxICDABEG* operon, which produces more 3OC6 and bioluminescence. The ArcA/ArcB two-component regulatory system responds to reducing conditions, and ArcA-P binds near the *lux* box, effectively inhibiting bioluminescence. Two other autoinducer pheromones AI-2 and C8-HSL are not shown, although the latter can also function with LuxR.

*Vibrio fischeri* is an attractive model for studying pheromone-mediated gene regulation and host-microbe symbiosis [40]. Bioluminescence in *V. fischeri* is regulated in part by the LuxR-LuxI pheromone system [28], and it is induced upon infecting the squid *Euprymna scolopes* in a natural and experimentally tractable symbiosis [40]–[42]. The *luxICDABEG* operon (Fig. 1) underlies bioluminescence and encodes the LuxI pheromone synthase, which produces *N*-(3-oxohexanoyl)-L-homoserine lactone (3OC6) [43]. When 3OC6 accumulates to a threshold concentration, it combines with LuxR to activate transcription of *luxICDABEG* [44]–[46]. Because the LuxI product 3OC6 induces *luxI* transcription, this “autoinducer” pheromone initiates a positive-feedback loop resulting in both increased 3OC6 production and bioluminescence.

The *V. fischeri lux* system also is regulated tightly in response to the environment, and such control is especially pronounced in isolates from *E. scolopes* such as strain ES114. ES114 is dim and produces little 3OC6 in culture, but in the host light organ it is ∼1000-fold more luminescent and produces more 3OC6 than in culture, even at similar high cell densities [47]–[49]. Several regulators modulate Lux expression [27], [38], [50], [51], perhaps none more impressively than the redox-responsive ArcA/ArcB two-component regulatory system [52]. ArcA is a direct repressor of *lux* and a *V. fischeri arcA* mutant is 100- to 1000-fold brighter than ES114 in culture, achieving nearly symbiotic luminescence levels [52].

In this study, we used *V. fischeri* and ArcA-mediated control of *lux* to examine the interplay between direct regulation by an environmentally-responsive regulator and the positive feedback inherent in pheromone production. We also explored the potential for intercellular signaling by distinct symbiotic *V. fischeri* populations. Our data illustrate important potential roles for 3OC6-mediated communication beyond sensing a quorum.

## Materials and Methods

### Media and Growth Conditions

*V. fischeri* was grown at 28°C or 24°C in one of three rich media depending on the application, as indicated below. The media used were LBS medium [53], ASWT medium [54], or SWTO medium [52]. *E. coli* strains were grown in either LB medium [55] or Brain Heart Infusion (Difco) at 37°C. Antibiotic selection for *V. fischeri* and *E. coli* strains was performed as described previously [56].

### Plasmid and Strain Construction

Bacterial strains, plasmids, and oligonucleotides used in this study are presented in Table 1. Plasmids were maintained in *E. coli* strain DH5α [57] except for plasmids that contained only the R6Kγ origin of replication (*oriV*_R6K_), which were maintained in strain DH5αλpir [56], or in strain CC118λpir [58] in the case of plasmid pEVS104. Plasmids that were stably maintained in *V. fischeri* were derived from shuttle vectors that contain both *oriV*_R6K_ and the replication origin from *V. fischeri* plasmid pES213 (*oriV*_pES213_) [56], [59]. These shuttle vectors were maintained in *E. coli* DH5αλpir prior to introducing them into *V. fischeri*. Plasmids were mobilized from *E. coli* into *V. fischeri* by triparental mating using CC118λpir pEVS104 as a conjugative helper and exploiting the RP4 origin of transfer (*oriT*_RP4_) as previously described [60].

**Table 1:** Strains, plasmids, and oligonucleotides used in this work.

Strains or Plasmids	Relevant characteristics^a^	Source or Reference	
**Strains**			
*Escherichia coli*			
DH5α	F’/*endA1 hsdR17 glnV44 thi-1 recA1 gyrA96* (Nx^R^) *relA1*	[57]	
	Δ(*lacIZYA-argF*)*U169deoR*(f80*dlacI*Δ(*lacZ*)*M15*)		
DH5αλpir	λ*pir* derivative of DH5α	[56]	
CC118λpir	Δ(*ara-leu*) *araD* Δ*lac74 galE galK phoA20 thi-1 rpsE rpsB argE*(Am) *recA* λ*pir*	[58]	
*Ralstonia solanacearum*			
AW1-AI8,395	*solI8*::SP *aidA395*::Tn*3*HoHo1 HSL-deficient Lac^+^ Nx^R^ Sp^R^ Cb^R^		[67]
*Vibrio fischeri*			
ES114	wild-type isolate from *E. scolopes* light organ		[47]
ANS3	ES114 Δ*luxI*; *luxI* 3OC6 synthase (VF_A0924) gene deletion		this study
ANS5	ES114 Δ*luxICDABEG*		this study
ANS6	ES114 Δ*arcA*::*erm* Δ*luxICDABEG*		this study
ANS7	ES114 Δ*arcA luxI* point mutant		this study
AMJ1	ES114 Δ*arcA*::*erm*; *arcA* (VF_2120) deleted and replaced		[52]
AMJ2	ES114 Δ*arcA*; *arcA* (VF_2120) gene deletion		[52]
CL21	ES114 *ainS*::*cat*; C8 synthase (VF_1037) mutant		[63]
EVS102	ES114 Δ*luxCDABEG*		[61]
JB33	ES114 Δ*arcA*::*erm* Δ*luxCDABEG*		this study
NL11	ES114 *ainS*::*erm litR*::*kan*		[50]
VCW2G7	ES114 *luxI* point mutant		[63]
**Plasmids**			
pAJ4	Δ*arcA* allele; *oriV*_R6Kγ_, *oriT*, Cm^R^		[52]
pAJ7	Δ*arcA*::*erm* allele; *oriV*_R6Kγ_, *oriT*, Cm^R^, Erm^R^		[52]
pAKD601B	*lacI^q^* and IPTG-inducible promoter, *oriV*_R6Kγ_, *oriV*_pES213_, *oriT*, Kn^R^		[81]
pAKD702	promoterless *lacZ*, *oriV*_R6Kγ_, *oriV*_pES213_, *oriT*, Cm^R^		[82]
pAS2	pEVS122 with *luxI* upstream sequence; *oriV*_R6Kγ_, *oriT*, Erm^R^		this study
pAS3	Δ*luxI* allele; *oriV*_R6Kγ_, *oriV*_ColE1_, *oriT*, Erm^R^, Kn^R^		this study
pAS4	Δ*luxICDABEG* allele; *oriV*_R6Kγ_, *oriV*_ColE1_, *oriT*, Em^R^, Kn^R^		this study
pAS6	Δ*arcA* allele (pAJ4) fused to pBluescript; *oriV*_R6Kγ_, *oriV*_ColE1_, *oriT*, Cm^R^, Amp^R^		this study
pAS104	pAKD601B-*arcA* (IPTG-inducible *arcA*), *oriV*_R6Kγ_, *oriV*_pES213_, *oriT*, Kn^R^		this study
pBluescript	*oriV*_ColE1_, Amp^R^		Stratagene
pEVS104	conjugative helper, *oriV*_R6Kγ_, *oriT*, Kn^R^		[60]
pEVS122	*oriV*_R6Kγ_, *oriT,* Erm^R^		[56]
pEVS148k	pCR-BluntII-TOPO with *luxI* downstream sequence, *oriV*_ColE1_, Kn^R^		[61]
pEVS149k	pCR-BluntII-TOPO with *luxG* downstream sequence, *oriV*_ColE1_, Kn^R^		[61]
pJLB169	Δ*arcA*::*erm* allele (pAJ7) fused to pBluescript; *oriV*_R6Kγ_, *oriV*_ColE1_, *oriT*, Cm^R^, Erm^R^, Amp^R^		this study
pJLB171	pAKD702 containing the *luxI* promoter region, *oriV*_R6Kγ_, *oriV*_pES213_, *oriT*, Cm^R^		[82]
pVSV102	*gfp*, *oriV*_R6Kγ_, *oriV*_pES213_, *oriT*, Kn^R^		[59]
pVSV208	*rfp*, *oriV*_R6Kγ_, *oriV*_pES213_, *oriT*, Cm^R^		[59]
**Oligonucleotides** b			
ASind_arcAF	ATGAGCTCTAACCAGTTAGTTAGGTACCG		this study
ASind_arcAR	TATCTAGAAAAGTCAGATAGAAGAGATTCTTA		this study
ASLUX1	CGGCTAGCCCATGCAACCTCTCTTATTTTACATGATC		this study
ASLUX2	ACCTGCAGGTGCGGTGAAGTTATTGAGCACAACATG		this study

a Kn^R^, Kanamycin resistance; Cm^R^ and *cat,* Chloramphenicol resistance; Erm^R^ and *erm,* Erythromycin resistance; Amp^R^, Ampicillin resistance; Nx^R^, Nalidixic acid resistance; Sp^R^, Spectinomycin resistance; Cb^R^, Carbenicillin resistance. Plasmid replication origins are designated *oriV* with a subscript indicating the source, and *oriT* indicates the RP4 origin of transfer.

b Oligonucleotides are in the 5′ to 3′ orientation with introduced restriction sites underlined.

To generate mutations in *V. fischeri*, mutant alleles were mobilized on unstable plasmids into recipients, and allelic exchange was screened using appropriate antibiotic resistance markers and PCR. To construct a Δ*luxI* mutant, sequence upstream of *luxI* was PCR amplified using primers ASLUX1 and ASLUX2 and cloned into pEVS122 at the SmaI site resulting in plasmid pAS2. pAS2 was fused at the NheI site to NheI-digested pEVS148k, which contains sequence downstream of *luxI,* resulting in the Δ*luxI* deletion construct pAS3. The Δ*luxI* allele on pAS3 was exchanged into ES114 to generate strain ANS3. To make the Δ*arcA luxI* double mutant strain ANS7, plasmid pAJ4 containing the Δ*arcA* allele was fused to pBluescript at their respective SpeI sites, resulting in plasmid pAS6, and the Δ*arcA* allele on pAS6 was exchanged into VCW2G7 (*luxI* point mutant). To construct the Δ*arcA*::*erm* Δ*luxCDABEG* strain JB33, the Δ*luxCDABEG* allele on plasmid pEVS153 was exchanged into the Δ*arcA*::*erm* strain AMJ1. To construct the *V. fischeri* Δ*luxICDABEG* mutant, pAS2 was fused at the NheI site to NheI-digested pEVS149k, which contains sequence downstream of *luxG,* resulting in the *luxICDABEG* deletion allele on plasmid pAS4. This Δ*luxICDABEG* allele was exchanged into ES114, generating strain ANS5. To make the Δ*arcA*::*erm* Δ*luxICDABEG* strain ANS6, the Δ*arcA*::*erm* allele on plasmid pJLB169 was exchanged into strain ANS5. To construct plasmid pAS104 with an inducible *arcA*, the *arcA* gene including 24 bp upstream of the ATG and 21 bp downstream of the TAA stop codon, was PCR amplified using primers ASind_arcAF and ASind_arcAR and directionally cloned into the SacI and XbaI sites of plasmid pAKD601B.

### Luminescence Assays

To assay luminescence, *V. fischeri* cultures were grown overnight in LBS medium and diluted 1∶1000 into either 25 ml SWTO medium in 125 ml flasks or 50 ml SWTO medium in 250 ml flasks. Within each experiment, the same flask and medium volume combinations were used for all strains and treatments. Media was supplemented with 2 mM isopropyl beta D-thiogalactopyranoside (IPTG) (Sigma-Aldrich, St. Louis, MO) or 50 nM 3OC6 (Sigma-Aldrich) where indicated. Cultures were incubated at 24°C with shaking at 200 rpm. At indicated time points, 0.5-ml samples were removed and the cell density was estimated by measuring the optical density at 595 nm (OD_595_) using a BioPhotometer (Brinkman Instruments, Westbury, NY). The cuvette was then shaken to aerate the sample and luminescence was measured using a GLOMAX 20/20 luminometer (Promega, Madison, WI) with a 10 sec integration setting. Luminescence values were normalized to cell density (OD_595_) unless indicated otherwise.

### ß-galactosidase Assays for Promoter Reporter Activity

*V. fischeri* strains harboring the P*_luxI_-lacZ* transcriptional reporter plasmid pJLB171 or the promoterless-*lacZ* vector parent pAKD702 were grown as described above for luminescence assays. At peak luminescence (OD_595_ ∼2.0–2.5) cells were collected by centrifugation and the supernatant was discarded. Cell pellets were frozen at −20°C overnight and ß–galactosidase assays were performed to determine Miller units as described previously [61].

### 3OC6 Bioassays

Previous studies assayed 3OC6 by adding samples to *V. fischeri* wild-type ES114, and endogenous 3OC6 production did not impede its utility in assaying exogenous 3OC6 [48], [62]. However, it has more recently been found that ES114 produces *N*-octanoyl homoserine lactone (C8) at much higher levels than 3OC6, and C8 can affect LuxR expression and activity [63]–[65]. For our bioassay we therefore used strain NL11 (Δ*ains*; *litR*::*kan*), which lacks both the C8 synthase (AinS) and the LitR regulator that modulates LuxR in response to C8 (or to the LuxS-generated AI-2).

To determine the level of 3OC6, *V. fischeri* cultures were grown in SWTO medium in aerobic shake flasks to an OD_595_ of 2.0 when cells were near peak luminescence. Cells were removed by centrifugation and the supernatant was extracted using equal parts supernatant and acidified ethyl acetate (1∶1000 acetic acid in ethyl acetate). The ethyl acetate layer was removed and allowed to evaporate in sterile glass flasks. After evaporation the extracted HSL was resuspended in a volume of SWTO equal to the original culture volume, and this extract-amended SWTO was inoculated 1∶1000 with *V. fischeri* bioassay strain NL11. Cultures were incubated at 24°C with shaking at 200 rpm, and cell density and luminescence were measured over time. Luminescence values for each sample extract were compared to those for 3OC6 standards to quantify the pheromone levels. For standards, 3OC6 (Sigma-Aldrich) was added into culture supernatant from a Δ*luxI* deletion strain (ANS3) to known concentrations (0, 1, 12.5, 25, 50, and 100 nM) and these 3OC6-amended supernatants were extracted and processed as described above.

### C8 Bioassays

C8 bioassays employed a *Ralstonia solanacearum* bioassay strain (AW1-AI8,395) that is responsive to unsubstituted HSLs with acyl chains of eight or more carbons [66], [67] and has been used previously to assay C8 levels in *V. fischeri* [27], [38], [66]. *V. fischeri* cultures were grown and HSLs extracted as described for the 3OC6 bioassays above. After evaporation the extracted HSL was resuspended in an equal volume of bioassay medium (per liter: 10 g tryptone, 5 g yeast extract, 0.5% glucose) and then diluted 1∶100 into 2 ml of bioassay medium. Strain AW1-AI8,395 was inoculated to an OD_595_ of ∼0.1 and tubes were incubated at 28°C with shaking at 200 rpm for ∼5 hr until cultures reached an OD_595_ of ∼0.4–0.6. Cells were collected to assay for ß–galactosidase activity as described previously [61] and Miller units for each sample extract were compared to those for C8 standards to quantify pheromone levels. For C8 standards, C8 (Sigma-Aldrich) was added to culture supernatant from a *V. fischeri ainS* mutant (CL21) to known concentrations (0, 1, 5, 10 nM) and these C8-amended supernatants were extracted and processed as described above. We used a Student’s t-test to determine whether strains had significantly different C8 accumulation.

### Squid Colonization Assays

*V. fischeri* cultures were grown in ASWT to an OD_595_ of 0.3–0.7 and diluted in Instant Ocean (United Pet Group Inc., Cincinnati, OH) to 660–3000 CFU ml^−1^. Aposymbiotic squid were added to the inoculum water overnight. The next morning squid were transferred to *V. fischeri*-free Instant Ocean. To measure the onset of luminescence in symbiotic animals, the luminescence per squid was measured using a LS6500 scintillation counter (Beckman Coulter, Fullerton, CA). At designated time points, squid were anesthetized with MgCl_2_, dissected and imaged using a Nikon (Melville, NY) Eclipse E600 epifluorescence microscope with a Nikon 96157 red filter cube, a Nikon 41017 green filter cube, and a Nikon Coolpix 5000 camera. After imaging, squid were homogenized and plated to determine CFU per squid.

## Results

### The arcA Mutant Produces High Levels of 3OC6 Pheromone

Previous work in *V. fischeri* showed *arcA* and *arcB* mutants have bright luminescence in culture relative to the parent strain [50], [52]. Since *luxI*, the 3OC6 synthase gene, is encoded in the same operon as the genes directly involved in generating bioluminescence, we predicted that an *arcA* mutant would also produce more 3OC6 pheromone than wild type. We found that at an OD_595_ of 2.0 when cultures are near peak luminescence, Δ*arcA* mutant cultures contained on average 55 nM 3OC6 pheromone while the wild-type, *luxI*, and Δ*arcA luxI* mutant cultures were below the level of detection for the assay (<1 nM). Thus, as predicted, ArcA mediates repression of not only bioluminescence but also 3OC6 pheromone synthesis.

### Bright Luminescence of the arcA Mutant is Mostly due to 3OC6-mediated Positive Feedback

**Figure 2:**
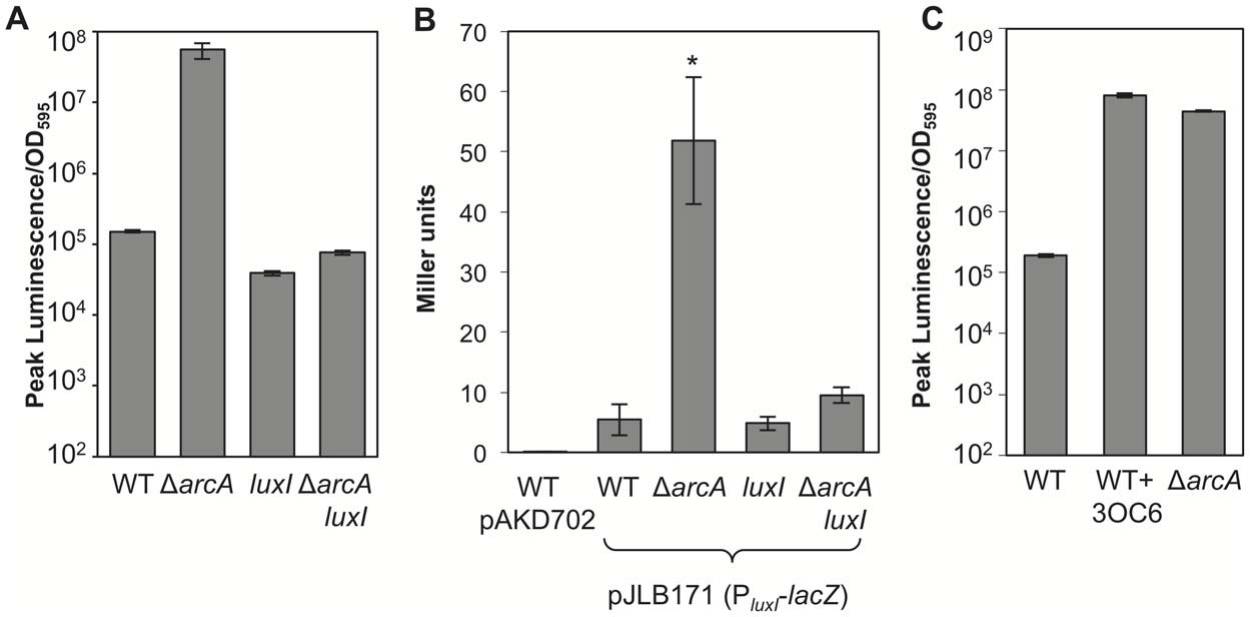
Effect of *luxI* and 3OC6 on derepression of the *lux* operon in an *arcA* mutant. Luminescence or *lux* reporter expression was measured in strains ES114 (WT), AMJ2 (Δ*arcA*), VCW2G7 (*luxI* mutant), or ANS7 (Δ*arcA luxI*). (A) Peak specific luminescence (luminescence per OD_595_) of strains grown in SWTO in aerobic shake flasks. Error bars indicate standard deviation (n = 3). (B) Plasmid based P*_luxI_*-*lacZ* transcriptional reporter assays. Strains containing pJLB171 or the promoterless vector pAKD702 were grown in duplicate aerobic shake flasks in SWTO medium. Cells were harvested at peak luminescence for ß–galactosidase assays. Error bars indicate standard deviation (n = 2). Asterisk indicates p-value of <0.05 with a Student’s t-test comparing the *arcA* mutant to its respective parent strain. (C) Peak specific luminescence values for aerobic cultures grown in SWTO medium with 50 nM 3OC6 added where indicated. Error bars (some too small to see) indicate standard deviation (n = 2). Each panel is representative of at least three independent experiments.

The ∼500-fold increase in luminescence in *arcA* mutants (documented previously [52]) should be a combined effect of the loss of direct, ArcA-dependent repression of the *lux* operon together with the 3OC6-mediated positive feedback inherent in the LuxR-LuxI regulatory circuit. To test the relative importance of these two effects we first assayed luminescence in a Δ*arcA* mutant strain with or without a functional *luxI* 3OC6 synthase gene. It is important to note that under these broth culture conditions, LuxR-mediated activation of the *lux* operon in ES114 is stimulated primarily by C8 [63], which is the product of AinS [68], [69]. C8 measurements ranged from 150 to 500 nM in different experiments but were never significantly different between ES114 and the Δ*arcA* mutant (p>0.2). The *arcA* mutant was >350-times more luminescent than wild type; however, in the *luxI* mutant background, the *arcA* mutation had only a 2-fold effect on luminescence (Fig. 2A). Similarly, we saw a much greater effect of the Δ*arcA* allele on a P*_lux_*-*lacZ* reporter in the wild-type background (10-fold) than in a *luxI* mutant (2-fold) (Fig. 2B). These results indicate that in the absence of ArcA-dependent repression of *lux*, bright bioluminescence is mediated primarily through the 3OC6-dependent positive feedback regulation of the *lux* genes.

To further explore the relative regulatory strengths of 3OC6-mediated positive feedback and direct repression of *lux* by ArcA, we tested whether addition of 3OC6 at concentrations found in cultures of the Δ*arcA* mutant could overcome ArcA-mediated repression of *lux* in wild-type cells. When 50 nM 3OC6 (the amount accumulated in *arcA* mutant cultures) was added to wild-type cultures, luminescence increased to levels comparable to that found in the Δ*arcA* mutant (Fig. 2C). Taken together, the results above suggested that ArcA-mediated repression of *lux* might be rendered inconsequential if cells have previously induced LuxI-mediated positive feedback or if a distinct population of neighboring cells lacks active ArcA-mediated repression of *lux*. Below we describe tests of both of these ideas.

### 3OC6-mediated Positive Feedback Results in Irreversible Luminescence Induction

**Figure 3:**
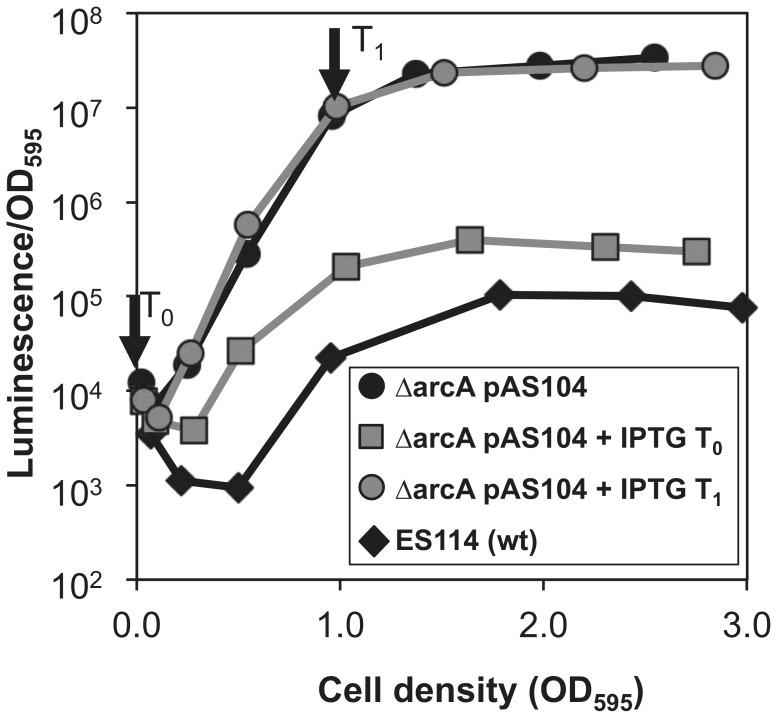
Effect of controlled ArcA expression on bioluminescence. Cultures of either the Δ*arcA* mutant with the inducible-*arcA* vector pAS104 (circles and squares) or wild type (diamonds) were grown in SWTO medium in duplicate aerobic shake flasks. Specific luminescence (luminescence per OD_595_) was observed for cultures grown with no addition (black) or with 2 mM IPTG added at the time of inoculation (T_0_, gray squares) or when cultures reached an OD_595_ of ∼1.0 (T_1_, gray circles).

Because an *arcA* mutant produces high levels of 3OC6 and exogenous 3OC6 can overcome ArcA-mediated *lux* repression, we asked whether ArcA can repress luminescence once the LuxI-mediated positive feedback circuit is initiated. To test this, we constructed an IPTG-inducible *arcA* expression vector (pAS104) and moved it into an *arcA* mutant to control when *arcA* is expressed. When the *arcA* mutant carrying pAS104 was grown in aerobic shake flasks without IPTG, luminescence reached the same high level observed when the empty vector was present (data not shown), suggesting ArcA expression in the absence of IPTG was low enough to have little regulatory impact. When IPTG was present from the start of the experiment (T_0_), luminescence was repressed to a level similar to that of the wild-type control (Fig. 3). However, when IPTG was added to cultures after luminescence was induced (T_1_), *arcA* expression was no longer able to repress luminescence, resulting in cultures with bright luminescence similar to that in the Δ*arcA* mutant without IPTG (Fig. 3). These results suggest that once 3OC6-mediated positive feedback is initiated fully by the loss of Arc-dependent repression, expression of ArcA cannot reverse this effect.

### 3OC6 from arcA Mutant Cells can Induce Luminescence in Neighboring Cells in Culture

**Figure 4:**
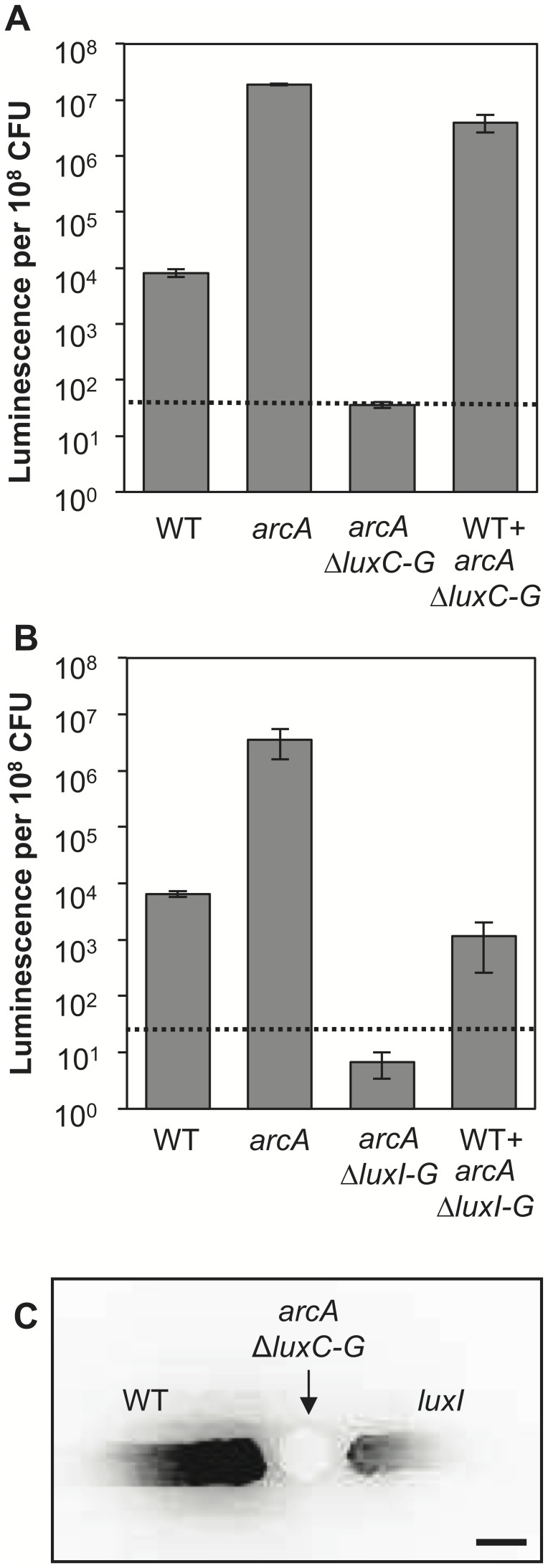
Inter-strain bioluminescence induction in mixed and spatially separated *arcA* ^+^ and *arcA* mutant cells. Wild-type cells were mixed with either the dark *arcA* Δ*luxCDABEG* strain (JB33) able to produce 3OC6 (A) or the dark *arcA* Δ*luxICDABEG* strain (ANS6) lacking the *luxI* 3OC6 synthase gene (B). Wild-type and *arcA* mutant cultures and co-cultures were grown in SWTO broth in duplicate aerobic shake flasks to peak luminescence. A sample was removed and dilution plated to determine the CFU ml^−1^ and the percent of wild-type cells in each co-culture (52% for A, 68% for B). Luminescence values are presented as luminescence per 10^8^ CFU. Luminescence values presented for co-cultures are luminescence per 10^8^ wild-type CFU. (C) Cultures of wild type or the *luxI* mutant were streaked onto SWTO agar plates next to spotted culture of the dark *arcA* Δ*luxCDABEG* strain (JB33) able to produce 3OC6. Inoculated plates were incubated at room temperature overnight and negative images of bioluminescence were captured with a BioRad Fluor-S MultiImager. Scale bar indicates approximately 5 mm.

We next asked whether 3OC6 from *arcA* mutant cells could induce luminescence in neighboring wild-type cells, overcoming ArcA-mediated repression of *lux* in the wild-type population. To examine this possibility we tested whether a dark (Δ*luxCDABEG*) *arcA* mutant could induce luminescence in wild-type cells in a 3OC6-dependent manner. When wild-type cells are co-cultured in the presence of dark (Δ*luxCDABEG*) *arcA* mutant cells in shake flasks, the specific luminescence of the wild-type cells increases nearly 500-fold (Fig. 4A), and this luminescence induction is dependent on *luxI* and 3OC6 pheromone production in the *arcA* mutant cells (Fig. 4B).

Additionally, we asked whether pheromone produced by the *arcA* mutant could be communicated across a distance when strains were spatially segregated. To test this we streaked wild-type and *luxI* mutant cultures onto an agar plate next to a spot of dark *arcA* mutant culture. After incubation, the wild-type streak showed high luminescence nearest the pheromone-producing *arcA* mutant, with elevated but diminishing luminescence in cells further from the pheromone source extending ∼15 mm (Fig. 4C). In contrast, the *luxI* mutant streak showed increased luminescence only nearest to the *arcA* mutant (Fig. 4C). The luminescence induction observed in both wild-type and *luxI* mutant streaks was dependent on *luxI* and 3OC6 synthesis in the dark *arcA* mutant (data not shown). The difference between luminescence of the wild-type and *luxI* streaks in Figure 4C presumably reflects induction in the *luxI* streak only by 3OC6 diffusing from the *arcA* mutant, whereas cells in the wild-type streak amplify the 3OC6 signal by positive feedback to extend the distance over which luminescence is induced. These results indicate that LuxI-mediated positive feedback considerably expands the range of this Arc-controlled signaling response.

### 3OC6 from One Population of Symbiotic Cells can Induce Luminescence in 3OC6-deficient Cells in the Squid Light Organ

Theoretically, inter-strain induction of luminescence such as that shown in Figure 4 could reflect a similar phenomenon in the light organ, with a subpopulation inducing luminescence in a broader population through amplification and diffusion of pheromone originating from a distinct subgroup. Therefore, we were interested in testing whether a subpopulation of symbiotic cells can induce luminescence in the wider community in the squid light organ. While the intercellular signaling experiments described above used *arcA* mutants as a subpopulation of cells with altered regulation of pheromone synthesis, we did not use *arcA* mutants in the squid colonization experiment described below. Previous studies showed wild-type *V. fischeri* cells produce high levels of pheromone in the light organ [48]. Moreover, ArcA does not repress luminescence in symbiotic cells [52], suggesting ArcA most likely does not repress pheromone synthesis in these cells as well. For these reasons, we used *arcA* ^+^ *V. fischeri* strains to test for intercellular signaling in the squid light organ.

**Figure 5:**
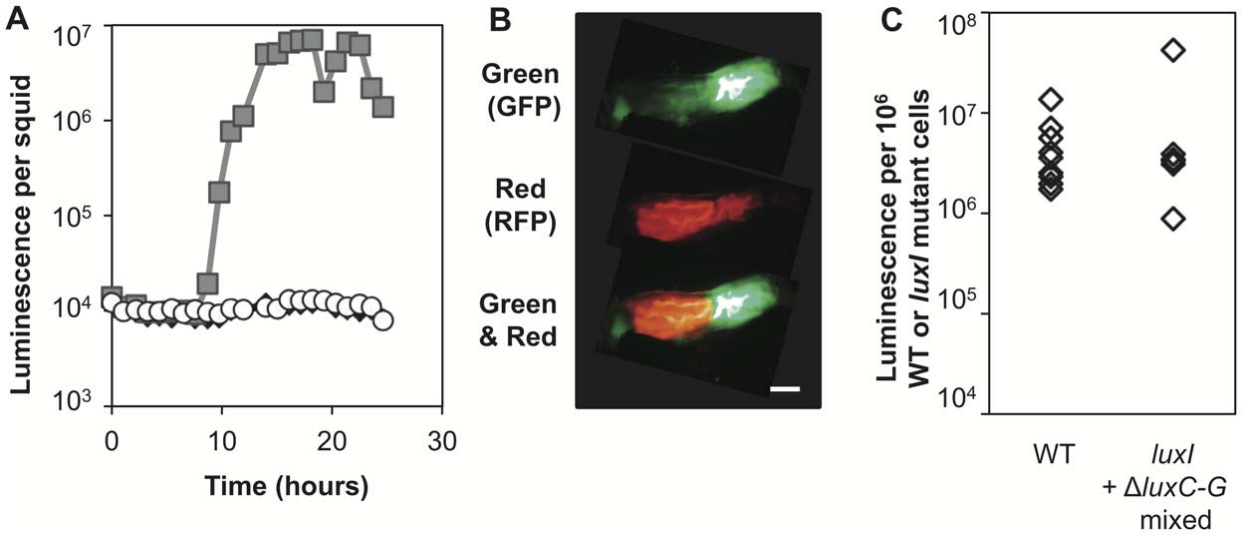
Inter-strain bioluminescence induction in the host light organ. (A) Relative luminescence induction over time for apo-symbiotic squid (black triangles) and squid infected with either the wild type (gray squares) or the VCW2G7 *luxI* mutant (empty circles). (B) GFP-labeled Δ*luxCDABEG* (EVS102) cells were mixed with RFP-labeled *luxI* mutant (VCW2G7) cells at a ratio of ∼1∶1 to inoculate apo-symbiotic juvenile squid. At 24 hr post infection, squid were dissected and imaged by epifluorescence microscopy to determine the spatial location of both cell types within the squid light organ using filters that allowed visualization of green fluorescence (top), red fluorescence (middle), or green and red fluorescence (bottom). Scale bar indicates approximately 100 µm. (C) Squid were then homogenized and plated to determine the CFU per squid for each cell type and to calculate the luminescence per 10^6^ wild-type or *luxI* mutant cells. Data include nine squid colonized with wild type and six co-colonized squid. Co-colonized squid contained 12–92% of the *luxI* mutant in the colonization. For reference, squid clonally colonized with the *luxI* mutant have an average background relative luminescence per 10^6^ CFU of <10^4^ on this scale.

To determine if a subpopulation of cells can induce luminescence in the wider light organ community, aposymbiotic juvenile squid were co-infected with a 1∶1 mixture of a *gfp*-labeled dark mutant (Δ*luxCDABEG*), that can still synthesize 3OC6, and a *rfp*-labeled *luxI* mutant that cannot make 3OC6. Neither strain alone can induce symbiotic luminescence [63] (Fig. 5A). However, when these strains co-colonize the light organ, if sufficient pheromone from the *gfp*-labeled dark mutant can diffuse into the pheromone deficient *rfp*-labeled strain, luminescence should be observed. 24 hr after infection, luminescence values were recorded for each squid before it was dissected and imaged with an epifluorescence microscope to visualize the spatial distribution of the two strains within the light organ (Fig. 5B). We observed wild-type levels of luminescence in the *rfp*-labeled *luxI* mutant cells in the squid when co-infecting the light organ with a dark 3OC6-producing strain (Fig. 5C). Interestingly, even when the dark pheromone-donor strain (Δ*luxCDABEG*) comprised as little as 8% of the light organ population in a mixed infection with the 3OC6-deficient (*luxI*) strain, the latter achieved luminescence comparable to that of wild-type in a clonal infection, and this robust induction of the *luxI* mutant by the dark strain occurred despite significant segregation of the two strains in the light organ. These results indicate that 3OC6-mediated intercellular signaling can occur between distinct subpopulations of symbiotic cells and that high levels of 3OC6 synthesis in only a subset of the population are sufficient to induce luminescence fully in the remaining cells occupying the light organ.

## Discussion

Bacterial pheromone signaling often is governed by environmental regulators and by pheromone-dependent positive feedback loops, both of which modulate the LuxR/LuxI pheromone signaling system that controls bioluminescence in *V. fischeri*. The relative strengths of environmentally responsive regulators and positive feedback have important implications for the biological functions of pheromone signaling in nature, and in this study we explored their contributions to *lux* regulation in the squid symbiotic strain ES114. We previously found that ArcA is a direct repressor of the *luxICDABEG* operon, and that *arcA* or *arcB* mutants are 100- to 1000-fold brighter than ES114 in culture [50], [52]. In this study we were surprised to find that in the absence of LuxI-mediated positive feedback, ArcA is actually a very weak repressor of the *lux* operon, repressing luminescence only about 2 fold. However, its influence over *lux* expression is amplified by LuxI-dependent positive feedback resulting in more than a hundred-fold additional increase in luminescence (Fig. 2).

Given the interest in mathematically modeling the *lux* circuit, we should note that our data do not necessarily indicate that a simple positive feedback circuit has such a large effect on *lux* operon transcription. Importantly for quantitative assessment of this circuit, luminescence output does not appear to correspond linearly with transcription from the *luxI* promoter. In studies either using *gfp* embedded in the *lux* operon or measuring transcripts by microarray analysis, conditions that induce luminescence 100- to 1000-fold only had a 10- to 20-fold effect on the GFP reporter or *lux* mRNA [52], [70], [71]. Similarly, in Figure 2 we show a ∼10-fold effect on expression of a plasmid-borne P*_luxI_*-*lacZ* reporter under conditions where luminescence was affected by at least an additional order of magnitude. Using the strain with *gfp* added to the *lux* operon, Perez *et al*. noted the relationship between fluorescence and luminescence followed a power law, and speculated that this may be due to the association equilibrium of LuxA and LuxB, which must dimerize to form active luciferase [71]. Unpublished data also suggest the *lux* operon may be post-transcriptionally controlled by a regulator that can be titrated with sufficient mRNA, which would also lead to a non-linear response between *lux* transcription and luminescence.

Given the overriding strength of positive feedback, we speculated that ArcA-mediated repression of *lux* would be rendered insignificant after a culture has engaged LuxI- (3OC6-) mediated positive feedback, or if *lux* in neighboring cells was highly expressed, for example following deactivation of the Arc system. Figures 3 and 4 illustrate support for these two ideas. Taken together these results have significant implications for the symbiotic role of pheromone signaling in at least two ways.

The first functional implication of our results is that the data in Figure 3 indicate an environmental condition in the host necessary for induction of 3OC6 signaling need not be maintained over time, because once the *lux* system is induced it essentially becomes more difficult to turn off. In the case of the ArcA/ArcB two component system, ArcB renders ArcA more active under reducing conditions and less active under oxidizing conditions. Bose *et al*. previously proposed that initial *V. fischeri* colonists might experience an oxidizing environment, leading to inactivation of ArcA/ArcB and induction of the *lux* operon but that later in infection, cells would become more crowded in the light-organ crypts, consume O_2_, and generate more reducing conditions that activate ArcA/ArcB [52]. They suggested that while ArcA might regulate other genes at this time later in infection, it would no longer effectively repress *lux* due to the increased synthesis of LuxI, accumulation of 3OC6, and positive feedback. More recently, studies by Williams et al. using transgenic *E. coli* strains also noted autoregulatory feedback by LuxR leading to more of this pheromone receptor in the cell and hysteresis [72], which may occur in strain ES114. The model proposed by Bose *et al*. reconciled the observations that ArcA did not repress *lux* in symbiotic cells but did appear to have a role in symbiont fitness later in infection [52]. The results of our study lend support for this model; however, our data also underscore the need to understand regulatory inputs during colonization of the host light organ. Given the discovery that direct regulation of the *lux* promoter by ArcA is quite weak, it is possible that the effect of another regulator is amplified by positive feedback and overpowers the repression by ArcA. The Arc activation state in symbiotic cells remains unknown, but as discussed below it is a priority for future research.

A second key implication of our results is that not all symbiotic cells would have to experience a stimulatory environment in the host for there to be a population-wide induction of luminescence. As noted above, high cell density alone will not induce luminescence in ES114, but the combination of a quorum and some aspect of the host environment foster induction of *lux* and bioluminescence. In Figure 4 we show that in the absence of ArcA-dependent repression of *lux*, 3OC6 can diffuse into nearby cells and induce luminescence despite ArcA actively repressing the *lux* operon in the cells receiving 3OC6. Superficially, the experiment shown in Figure 4C resembles many others wherein added pheromone or a pheromone-producing strain stimulates a response in cells lacking pheromone production, with the signal diffusing over millimeter distances [73], [74]. However, the key difference is that in Figure 4C an environmentally responsive regulator is what distinguishes pheromone levels in the adjacent strains. In short, Figure 4 shows that in a heterogeneous population, either mixed or spatially segregated, derepression of pheromone synthesis and the ensuing positive feedback override direct repression. These results imply that a group response can be coordinated based on physiological conditions that are not necessarily being experienced by the entire population.

**Figure 6:**
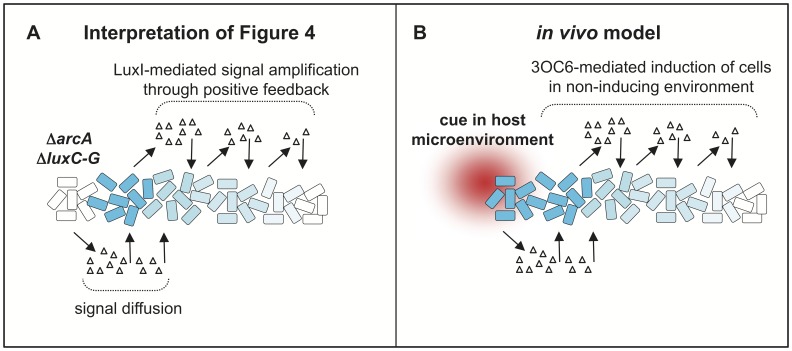
Models of how subpopulations with heterogeneous Arc status could coordinate population-wide responses. Triangles indicate 3OC6 pheromone, white cells indicate no luminescence, and blue cells indicate luminescent cells. Panel A illustrates an interpretation of the data in Figure 4C, and panel B illustrates how similar signaling might occur in the host environment. In panel B we propose that even if only a subset of cells in the light organ experience physiological changes that silence ArcB kinase activity, this could result in loss of ArcA-dependent repression of *lux* followed by increased production of 3OC6 pheromone signals. This 3OC6 could then diffuse into neighboring ArcA-repressed cells to initiate positive feedback regulation of *lux,* thereby inducing luminescence in a population-wide response to conditions sensed by ArcB in a sub-population of symbionts.

Figure 6 further illustrates how we interpret the data shown in Figure 4C, and how this result could reflect luminescence induction in the symbiosis. In our model, some environmental cue in a host microenvironment is found in a gradient. For example, if oxygen, reactive oxygen species or another good electron acceptor were provided by the host but also consumed by the bacteria (e.g. oxygen consumed by luciferase), there could be a relatively steep gradient of this cue. Perhaps symbionts nearer the host epithelium are exposed to a sufficiently oxidative environment to turn off Arc while others deeper in the crypt lumen experience more reduced conditions wherein Arc is active. The first group, with inactive Arc, might then initiate LuxI-mediated pheromone synthesis, producing a signal that spreads through the population. Quite the opposite of the original environmental cue, the pheromone signal would not be destroyed by other symbiont cells but rather it would be amplified by positive feedback, leading to a much more shallow gradient. Put together, this scenario could allow cell-cell signaling to induce a broader population response based on an environment only experienced by a subpopulation.

Our model in Figure 6B in part invokes a scenario wherein 3OC6 signal from a subpopulation in the light organ initiates a population-wide response, and while this seemed plausible to us it had never been tested. In Figure 5, we observed pheromone-mediated intercellular communication in the squid light organ, where dark 3OC6-producing cells induced luminescence in co-colonizing, 3OC6-deficient cells. This inter-strain signaling in the light organ was particularly remarkable in that only a relatively small and segregated portion of the population needed to be producing 3OC6 in order to induce luminescence fully in the remaining cells. Moreover, it is worth noting that only the 3OC6-producing cells retain positive feedback regulation of pheromone synthesis, and therefore such positive feedback was not required for the subpopulation receiving the signal to induce luminescence fully. These data are proof in principle that symbiotic luminescence induction could originate from a regulatory response by a subpopulation. We have previously shown that symbiotic *lux* induction is spatially heterogeneous, indicating that subpopulations in different light organ microenvironments experience different regulatory cues [59]. The regulators and environmental conditions underlying this heterogeneity, and whether in fact subpopulations are responsible for the ultimate population-wide induction of luminescence, are fertile areas for future research.

Pheromone production is required for luminescence induction in *V. fischeri* cells colonizing the squid; however, as noted above the environmental cues and regulators responsible for activating symbiotic pheromone synthesis and luminescence remain uncertain. Previous work showed that *arcA* mutant cells are brighter than wild type in culture but not in the squid [52], consistent with a model in which inactivation of ArcA/ArcB in the host may be important for luminescence derepression in symbiotic cells. Future work will focus on directly testing the role of ArcA/ArcB in symbiotic luminescence induction in *V. fischeri* and on elucidating the physiological conditions that control the activity of this two-component system in *V. fischeri*. Studies of *E. coli* have indicated that oxygen itself is not perceived by ArcA/ArcB [75], and varied reports have suggested that ArcB may sense and respond to fermentation acids [76], [77] and/or the redox state of the quinone pool [78], [79]. The predicted ArcA regulon in *V. fischeri* [80] appears to be very similar to that of *E. coli* [80], and the *V. fischeri arcA* gene complements an *E. coli arcA* mutant [52], suggesting functional conservation of this two-component system across these two bacterial species. However, the mechanism underlying ArcB sensing of redox state in *V. fischeri* remains uncertain. Future work in this area will elucidate both how Arc functions and how it is integrated with pheromone signaling in a natural infection.

Our results are also consistent with a different regulatory input overpowering ArcA-mediated *lux* repression. Figure 2C directly illustrates that 3OC6 can induce luminescence and render ArcA-mediated *lux* repression insignificant. This result shows that if another regulator induces 3OC6-mediated positive feedback in symbiotic cells, whether or not Arc is repressing *lux* may not matter. We are actively investigating the Arc status of symbiotic *V. fischeri* cells, but we are also elucidating regulators other than Arc that could play key roles in symbiotic pheromone signaling. A recent mutant screen identified transposon-insertion mutants in thirteen loci other than *arc* that led to increased luminescence of ES114 in culture [50]. These and other regulatory inputs should also be considered with respect to *lux* induction in the symbiotic environment.

The *lux* pheromone system in *V. fischeri* is controlled by environmental regulators, and we have shown that such inputs can be powerfully amplified by the positive feedback inherent in the *lux* circuitry. Because bacterial pheromones are often regulated in response to environmental conditions and also subject to positive feedback, we expect that these results may reflect signaling systems in many other host-associated bacteria. Although pheromone signaling may require a sufficiently high cell density for effective communication, the pheromones cannot be considered simply as census-taking molecules. Scenarios similar to the model presented in Figure 6B may explain the combination of environmental regulation and positive feedback in the pheromone signaling systems of host-associated bacteria, and this possibility should be considered as we seek to understand the biological roles of these systems.
